# Avoiding holiday seasonal weight gain with nutrient-supported intermittent energy restriction: a pilot study

**DOI:** 10.1017/jns.2019.8

**Published:** 2019-03-25

**Authors:** Steven P. Hirsh, Marianne Pons, Steven V. Joyal, Andrew G. Swick

**Affiliations:** 1 Life Extension Clinical Research, Inc., 5990 North Federal Highway, Fort Lauderdale, FL 33308, USA; 2 Life Extension, Inc., 3600 West Commercial Boulevard, Fort Lauderdale, FL 33309, USA

**Keywords:** Body weight, Modified 5:2 diet, Intermittent energy restriction, Dietary supplements, Winter holiday, Insulin, Lipid profile, ALT, alanine aminotransferase, AST, aspartate aminotransferase, HOMA2, updated homoeostasis model assessment

## Abstract

This pilot randomised controlled study evaluated the effects of a nutrient-supported intermittent energy restriction nutrition programme to prevent weight gain in healthy overweight adults during the 6-week winter holiday period between Thanksgiving and New Year. For 52 d, twenty-two overweight adults (mean age 41·0 years, BMI 27·3 kg/m^2^) were assigned to either the nutrition programme (*n* 10; two fasting days of 730 kcal/d (3050 kJ/d) of balanced shake and dietary supplements to support weight management efforts, followed by 5 d of habitual diet) or a control group (*n* 12; habitual diet). A significant weight loss from baseline (pre-holiday 10 d before Thanksgiving) to day 52 (post-holiday 3 January) was observed in the nutrition programme (75·0 (sd 9·8) *v.* 76·3 (sd 9·8) kg; *P* < 0·05). Body weight did not significantly change in the control group and there was no between-group difference. Increases from baseline in fasting insulin (42·9 %; *P* = 0·0256), updated homoeostasis model assessment (HOMA2) (43 %; *P* = 0·025), LDL-cholesterol (8·4 %; *P* = 0·0426) and total cholesterol (7·1 %; *P* = 0·0154) levels were also reported in the control group. In the nutrition programme group, baseline HDL-cholesterol and TAG levels measured after two fasting days increased (13 %; *P* = 0·0245) and decreased (22·8 %; *P* = 0·0416), respectively. There was no significant change in HOMA2. Between-group differences in changes in insulin levels (*P* = 0·0227), total cholesterol:HDL-cholesterol ratio (*P* = 0·0419) and HOMA2 (*P* = 0·0210) were significant. Overall compliance rate was 98 % and no severe adverse events were reported. These preliminary findings suggest that this intermittent energy restriction intervention might support weight management efforts and help promote metabolic health during the winter holiday season.

Global obesity rates have been steadily rising over the last 30 years and are reaching epidemic proportions. Although weight gain generally occurs over time, some periods are considered especially problematic. The winter holiday season, a traditional feasting time ingrained in many Western cultures, is one of them. Depending on sample composition and study design, several studies conducted during the holiday season in US adults consistently reported increases in body weight of 0·37 kg^(^^1^^)^ and 0·78 kg^(^^2^^)^ between mid-November and early/mid-January, 0·4 kg over the Christmas period^(^^3^^)^, 0·5 kg during a 13-d period over Thanksgiving^(^^4^^)^, and 0·6 kg between Christmas and New Year^(^^5^^)^. Evidence shows weight gain during the 6-week holiday season between Thanksgiving and the New Year is a significant contributor to annual weight gain for many^(^^1^^)^. Similar trends have been described in other countries. In the UK, studies found mean 0·9 kg weight gains between Christmas and early/end January^(^^6^^,^^7^^)^. A study involving wireless digital scales with 760 participants in Germany reported a 0·8 kg weight gain between Christmas and New Year^(^^5^^)^. This weight gain is often maintained into the summer months and beyond^(^^1^^,^^5^^)^, and, if repeated every year, may result in cumulative weight gain over time. Data also show that overweight individuals tend to gain more weight than normal-weight individuals^(^^1^^,^^4^^)^. Steady weight gain over the years may lead to obesity, which is a risk factor for many chronic diseases and increased morbidity and mortality^(^^8^^–^^12^^)^. Evidence also shows that obesity greatly impacts quality of life and mental health^(^^13^^)^. Importantly, even a modest 5 % weight loss is enough to produce substantial health improvements^(^^14^^,^^15^^)^. Therefore, effective dietary strategies need to be implemented to prevent weight gain during the sensitive winter holiday season. Yet, very few studies have investigated such strategies. Some have explored cognitive–behavioural treatment and self-monitoring interventions or supplementation with conjugated linoleic acid^(^^16^^,^^17^^)^, but none has evaluated the benefits of intermittent fasting.

The winter holiday season is a time for celebration often associated with an overindulgence of delicious, highly palatable, and readily available foods usually rich in fats and sugars. Along with a more sedentary lifestyle during the cold autumn and winter months, increased energy intake and decreased energy expenditure may promote weight gain if this episode of energy imbalance is maintained. Many different strategies have been developed over the years that could be effective approaches for managing holiday weight gain. Long-term energy restriction is challenging and unrealistic during the holiday season for many individuals. On the other hand, intermittent fasting – short periods of energy intake reduction significantly below the amount normally consumed between longer periods of habitual energy intake – has become increasingly popular in the lay press and has been shown to result in weight loss and numerous health benefits^(^^18^^–^^28^^)^. A well-known short-term intermittent fasting approach is the popular 5:2 diet. Clinical studies have demonstrated the many health benefits associated with consuming 500–650 kcal/d (2090–2720 kJ/d) on two scheduled fasting days a week, and *ad libitum* eating for the other 5 d in overweight and obese adults^(^^22^^,^^24^^,^^29^^)^. This is an attractive strategy for weight management during the holiday season, as reduced energy intake during fasting days may offset excessive energy intake during *ad libitum* days, without ‘calorie counting’ or food restriction. Yet, no study has addressed the benefits of a 5:2 intermittent fasting approach during the winter holiday season.

Based on the above considerations, this pilot, single-centre, parallel-group, randomised and controlled study was designed to evaluate the effects of a modified 5:2 intermittent energy restriction nutrition programme to prevent weight gain in overweight healthy adults over the winter holiday period, compared with a control group following their habitual diet.

## Materials and methods

### Participants

**Fig. 1.**
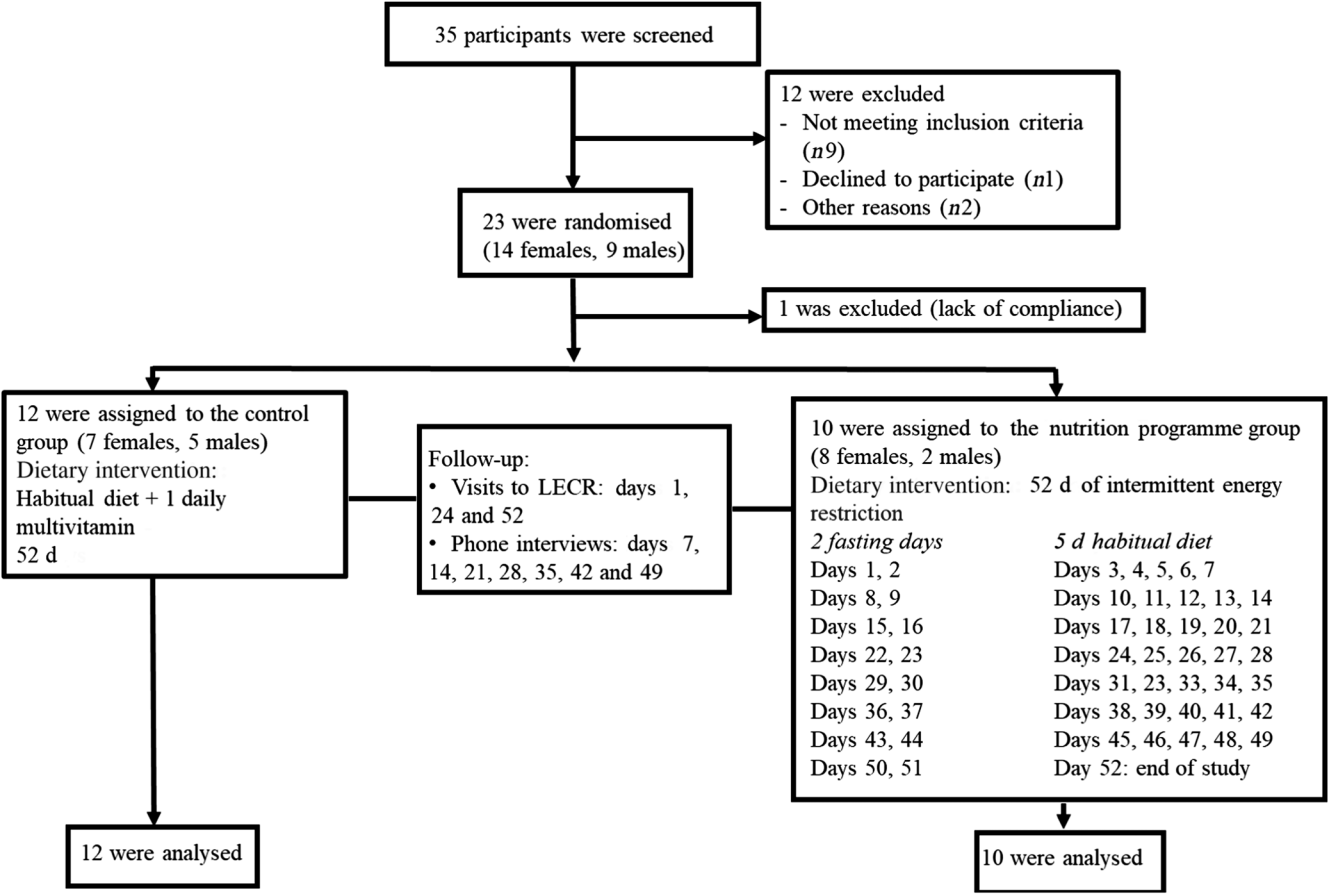
Consolidated Standards of Reporting Trials (CONSORT) flow diagram for study participants. LECR, Life Extension Clinical Research.

Subjects were recruited by means of posters and flyers, advertisement on social media, and email blasts to potential volunteers in the Life Extension database. We did not perform an *a priori* sample size calculation as results from this pilot trial will be used to conduct power analyses for a future larger study. Eligible participants were overweight healthy males and females, aged 21-65 years with a BMI between 25 and 29·9 kg/m^2^. Having a stable weight (±3 kg) over the past 6 months preceding the study, no known or suspected gastrointestinal disorders, and no difficulty swallowing (dysphagia) or difficulty chewing were among the inclusion criteria. Exclusion criteria included having been diagnosed with, received medical treatment for, or taking daily medication for type 2 diabetes, dyslipidaemia, hypertension, impaired glucose tolerance, impaired fasting glucose, and other metabolic diseases. A signed informed consent was obtained from each participant. Of thirty-five subjects assessed for eligibility, twenty-three were randomised. One female subject in the nutrition programme group was excluded after the first week due to lack of compliance; therefore, twenty-two participants (thirteen females, nine males) completed the study and were included in the final analyses (Fig. 1). The study was conducted according to the Declaration of Helsinki. The study was approved by IntegReview Institutional Review Board and registered at ClinicalTrials.gov (NCT03372109).

### Dietary interventions

The nutrition programme consisted of 52 d of intermittent energy restriction. On two consecutive days (Monday and Tuesday) of each week, participants decreased their energy intake (730 kcal/d; 3050 kJ/d) by consuming a commercially available shake (170 kcal (710 kJ/d); 12, 6, 24 and 24 % of total energy from fat, carbohydrate, fibre and protein, respectively) (Life Extension) four times per d (in the morning, at lunch, in the afternoon, and at dinner). On the remaining 5 d (Wednesday to Sunday) participants were instructed to eat their habitual diet with no specific dietary recommendations. The nutrition programme also included the daily consumption of a set of commercially available dietary supplements (Life Extension) selected not only for their potential overall health benefits, but to ensure adequate intake of key essential nutrients, including vitamins and minerals, and support weight management efforts. Ingredients in the supplements included long-chain *n*-3 fatty acids (EPA and DHA)^(^^30^^,^^31^^)^ found in fish oil (this supplement provided an additional 50 kcal/d (210 kJ/d)), sesame lignans^(^^32^^)^, olive (*Olea europaea*) fruit and leaf extract^(^^33^^)^, plant-based polyphenols, saturated fats, nuts and olive extract to mimic the Mediterranean diet^(^^34^^)^, soluble fibres (xylooligosaccharides)^(^^35^^)^, Italian Borlotto variety of white kidney bean (*Phaseolus vulgaris*)^(^^36^^,^^37^^)^, saffron^(^^38^^)^, clove bud (*Syzygium aromaticum*) and maqui berry (*Aristotelia chilensis*) extracts^(^^39^^–^^42^^)^, curcumin^(^^43^^–^^45^^)^, coenzyme Q_10_–ubiquinol and shilajit^(^^46^^)^, *Gynostemma pentaphyllum* extract^(^^47^^,^^48^^)^ and hesperidin^(^^49^^)^. Participants in the control group were required to follow their habitual diet without any restriction and take one tablet of a commercially available multivitamin daily for 52 d. All subjects were instructed to drink at least eight cups of water/d and engage in their typical physical activity.

### Study design and procedures

This 52-d, single centre, parallel-group, randomised and controlled trial (13 November–3 January) was conducted at Life Extension Clinical Research (LECR) (Fort Lauderdale, FL). Subjects were randomly assigned by blocks of four to either the nutrition programme or control group. Three visits were scheduled at LECR throughout the holiday season. The initial pre-holiday visit took place on 13 November (day 1; baseline), 10 d before Thanksgiving. Subjects were brought back for a second mid-holiday visit (day 24) on 6 December (between Thanksgiving and Christmas) and a third and final visit (day 52) on 3 January (post-holiday). Assessments were conducted in the morning following a 12 h fast on days 24 and 52 in the control group, and in the morning after the two fasting days in the nutrition programme group. Regular telephone interviews and email communications were conducted to discuss any change in medical history or concomitant medications, monitor adverse events, reinforce compliance, and remind subjects of the date and time of their next visit. For both groups, the allowable window was +1 d for visits 3 (day 24) and 4 (day 52), and −3 d for scheduled telephone interviews. Body weight was measured using a DIGI DS-160 calibrated scale (Rice Lake Weighing Systems) with no shoes on. Blood pressure and heart rate were obtained with a Digital Blood Pressure Monitor Model UA-767 Plus device (LifeSource). Blood pressure was measured in duplicate after 10 min at rest while subjects were in a seated position, and the mean value was calculated. A venous blood sample was also drawn. Immediately following collection, tubes containing K2 EDTA were gently inverted up to eight times to ensure proper mixing before centrifugation (10 min at 2500 rpm). Serum was promptly collected, and specimens were refrigerated until shipped to LabCorp for processing. Insulin was measured using a two-site electrochemiluminescent immunoassay on the Elecsys 1010/2010 and MODULAR ANALYTICS E170 automated platform (Roche Diagnostics Corporation). Enzymic methods (Roche Diagnostics Corporation) were used to assess glucose (assay no. 05168791), total cholesterol (assay no. 05168538), LDL (assay no. 07005768), HDL (assay no. 07528582), TAG (assay no. 05171407), aspartate aminotransferase (AST) (assay no. 05850819) and alanine aminotransferase (ALT) (assay no. 05850797) levels on a Roche/Hitachi Cobas c 701/702 automated analyser (Roche Diagnostics Corporation).

### Primary outcome assessment

Within- and between-group absolute changes from baseline in body weight were assessed at days 24 and 52.

### Secondary outcome assessments

#### Serum metabolic markers

Within- and between-group absolute changes from baseline in fasting insulin, LDL-cholesterol, HDL-cholesterol, total cholesterol and TAG levels were evaluated at day 52. Changes in insulin sensitivity were estimated at day 52 using the updated homoeostasis model assessment (HOMA2)^(^^50^^–^^52^^)^. Lower HOMA2 values indicated higher insulin sensitivity, and higher HOMA2 values indicated lower insulin sensitivity, and therefore suggested greater insulin resistance.

#### Compliance

At each visit at days 24 and 52, subjects in the nutrition programme were asked to return containers to evaluate remaining study product, discuss adherence, and calculate compliance. Subjects were counselled when compliance was less than 85 %. Participants were asked to not transfer the study product from the original container to another container.

#### Safety

Within- and between-group absolute changes from baseline in vital signs (systolic blood pressure, diastolic blood pressure and heart rate) and liver blood chemistry (AST and ALT) were assessed at day 52. Participants were asked to contact LECR immediately if experiencing adverse effects. Symptoms and signs of adverse events were documented throughout the study.

### Statistical analysis

Statistical analysis was performed using GraphPad Prism software, version 5.01 (Abacus Concepts GraphPad Software) and SAS statistical software, version 9.4 (SAS Institute Inc.). Results in tables are presented as means and standard deviations. The Shapiro–Wilk test was used to assess normal distribution of the data sets. For the primary outcome, within-group absolute changes were analysed with one-way repeated-measures ANOVA and between-group absolute changes with one-way independent-variables ANOVA, with Bonferroni's multiple comparison post-test. For secondary outcomes, paired Student's *t* test or Wilcoxon matched pairs test were used to analyse within-group changes, and unpaired Student's *t* test or Mann–Whitney *U* test were used to analyse between-group changes. Unpaired Student's *t* test was used to evaluate baseline differences between groups. Pearson correlation coefficients (*r*) were calculated to evaluate the correlation between baseline body weight and changes in body weight at day 52 in both groups. The level of significance was set at *P* < 0·05.

## Results

### Clinical characteristics at baseline

Results are shown in Table 1. In all, twenty-two subjects completed the study (nutrition programme group, *n* 10 (eight females, two males) and control cohort, *n* 12 (five females, seven males)) and were included in the final analyses. Mean age of all participants was 41·0 years, mean body weight was 78·0 kg, and mean BMI was 27·3 kg/m^2^. There was no statistically significant difference in clinical pre-holiday baseline (before Thanksgiving) characteristics between groups.

**Table 1.** Clinical characteristics of study participants at baseline (pre-holiday on 13 November, 10 d before Thanksgiving) (Mean values and standard deviations)

	Nutrition programme group (*n* 10)		Control group (*n* 12)		All (*n* 22)	
Characteristics	Mean	sd	Mean	sd	Mean	sd
Age (years)	43·4	13·0	39·0	10·7	41·0	11·7
Weight (kg)	76·3	9·8	79·4	8·9	78·0	9·3
BMI (kg/m^2^)	26·7	1·9	27·7	3·1	27·3	2·6
Systolic blood pressure (mmHg)	108·2	6·4	114·6	12·2	111·7	10·3
Diastolic blood pressure (mmHg)	72·9	4·7	75·7	7·3	74·4	6·3
Heart rate (beats/min)	66·7	7·8	72·4	8·0	69·8	8·3

### Primary outcome

Results are shown in Table 2. After 24 d, subjects in the nutrition programme group started losing weight (75·3 (sd 9·5) *v.* 76·3 (sd 9·8) kg), although this was not statistically significant. After 52 d, participants lost a total of 1·3 kg (1·7 %) from baseline (75·0 (sd 9·8) *v.* 76·3 (sd 9·8) kg; *P* < 0·05). Subjects in the control group lost 0·3 kg from baseline at day 24 and 0·4 kg at day 52, which was not statistically significant. There was no significant between-group difference in weight loss at day 24 or day 52. When classified based on sex, males in the nutrition programme group (*n*  2) lost significantly more weight at day 24 than males in the control group (*n*  7) (−1·9 (sd 0·7) *v.* −0·5 (sd 0·7) kg; *P* = 0·0481), while no difference was found in females. No difference was found at day 52 for either males or females. No significant correlation was found between baseline body weight and changes in body weight at day 52 for subjects in the control group (*r* −0·37; *P* = 0·2365) or nutrition programme group (*r* −0·09; *P* = 0·8001).

**Table 2.** Body weight in the nutrition programme group (*n* 10) and control group (*n* 12) at baseline (pre-holiday on 13 November, 10 d before Thanksgiving), day 24 (mid-holiday on 6 December, between Thanksgiving and Christmas) and day 52 (post-holiday, 3 January) (Mean values and standard deviations)

	Baseline		Day 24		Absolute change at day 24		Day 52		Absolute change at day 52	
	Mean	sd	Mean	sd	Mean	sd	Mean	sd	Mean	sd
Nutrition programme group	76·3	9·8	75·3	9·5	−1·0	0·9	75·0*	9·8	−1·3	1·8
Control group	79·4	8·9	79·1	8·4	−0·3	0·9	79·0	8·5	−0·4	1·4

* Mean value was significantly different from that at baseline (*P* < 0·05; within-group with one-way repeated-measures ANOVA and Bonferroni multiple comparison post-test).

### Secondary outcomes

#### Serum metabolic markers

Results are shown in Table 4. There was no significant difference at baseline between groups. After a 12 h fast in the control group, there was a significant 49·2 % increase compared with baseline in insulin (10·0 (sd 6·5) *v.* 7·0 (sd 3·2) *μ*IU/ml (69·5 (sd 45·1) *v.* 48·6 (sd 22·2) pmol/l); *P* = 0·0256), 8·4 % increase in LDL-cholesterol (121·1 (sd 35·7) *v.* 111·7 (sd 29·9) mg/dl (3·1 (sd 0·9) *v.* 2·9 (sd 0·8) mmol/l); *P* = 0·0426), and 7·1 % increase in total cholesterol (196·8 (sd 48·1) *v.* 183·6 (sd 41·0) mg/dl (5·1 (sd 1·2) *v.* 4·8 (sd 1·1) mmol/l); *P* = 0·0154) levels after 52 d. Between-group difference in changes from baseline in insulin levels and total cholesterol:HDL-cholesterol ratio was significant (*P* = 0·0227 and *P* = 0·0419, respectively). In the nutrition programme group, there was a significant 13 % increase in HDL-cholesterol (70·0 (sd 21·0) *v.* 62·0 (sd 18·0) mg/dl (1·8 (sd 0·5) *v.* 1·6 (sd 0·5) mmol/l); *P* = 0·0245) and a significant 22·8 % reduction in TAG levels (70·5 (sd 34·8) *v.* 91·9 (sd 46·6) mg/dl (0·8 (sd 0·4) *v.* 1·0 (sd 0·5) mmol/l); *P* = 0·0416) compared with baseline after the two fasting days. In addition, there was a significant 43 % increase in HOMA2 in the control group after 52 d compared with baseline (1·33 (sd 0·86) *v.* 0·93 (sd 0·43); *P* = 0·025) *v.* no significant change in the nutrition programme group; however, there was a trend towards a decline (0·73 (sd 0·49) *v.* 0·91 (sd 0·45)) seen in the nutrition programme group. The between-group difference in change from baseline in HOMA2 was statistically significant (−0·18 (sd 0·27) in the nutrition programme group *v.* 0·40 (sd 0·53) in the control group (*P* = 0·0041).

#### Compliance

Compliance with the nutrition programme group was very good throughout the study (98·0 (sd 7·3) %), with similar rates of adherence at day 24 (97·9 (sd 10·3) %) and day 52 (98·0 (sd 6·5) %).

#### Safety: vital signs

There was no significant change in systolic blood pressure, diastolic blood pressure and heart rate in either the nutrition programme or control group throughout the study (Table 3).

**Table 3.** Vital signs in the nutrition programme group (*n* 10) and control group (*n* 12) at baseline (pre-holiday on 13 November, 10 d before Thanksgiving) and day 52 (post-holiday, 3 January) (Mean values and standard deviations)

	Baseline		Day 52		Absolute change	
Parameters	Mean	sd	Mean	sd	Mean	sd
Systolic blood pressure (mmHg)						
Nutrition programme group	108·2	6·4	108·2	11·6	0·0	12·7
Control group	114·6	12·2	114·3	12·5	−0·3	10·7
Diastolic blood pressure (mmHg)						
Nutrition programme group	72·9	4·7	73·3	7·3	0·4	7·0
Control group	76·0	7·0	77·0	6·0	1·0	6·2
Heart rate (beats/min)						
Nutrition programme group	66·7	7·8	62·0	6·3	−4·7	8·6
Control group	72·4	8·0	72·1	5·6	−0·3	4·3

#### Safety: liver blood chemistry

Results are shown in Table 4. At baseline, all measured serum parameters were within normal range in both groups. However, ALT levels were significantly higher in the control group than the nutrition programme group (25·5 (sd 14·0) *v.* 15·5 (sd 4·5) IU/l; *P* = 0·0428). In addition, although ALT levels significantly increased in the nutrition programme group after 52 d compared with baseline, they were still within normal limits (21·2 (sd 8·2) *v.* 15·5 (sd 4·5) IU/l; *P* = 0·0278). Of note, ALT levels also increased in the control group from 25·5 (sd 14·0) IU/l at baseline to 29·8 (sd 20·2) IU/l at day 52, although not significantly. AST levels did not significantly change.

**Table 4.** Liver blood chemistry and fasting serum metabolic markers in the nutrition programme group (*n* 10) and control group (*n* 12) at baseline (pre-holiday on 13 November, 10 d before Thanksgiving) and day 52 (post-holiday, 3 January) (Mean values and standard deviations)

	Baseline		Day 52		Absolute change	
Parameters	Mean	sd	Mean	sd	Mean	sd
Liver blood chemistry						
ALT (IU/l)						
Nutrition programme group	15·5‡	4·5	21·2*	8·2	5·7	6·9
Control group	25·5	14·0	29·8	20·2	4·3	10·9
AST (IU/l)						
Nutrition programme group	18·1	3·2	20·7	6·1	2·6	5·1
Control group	22·8	9·6	24·7	11·2	1·9	5·6
Metabolic markers						
Insulin (*μ*IU/ml)‖						
Nutrition programme group	6·9	3·4	6·4	3·7	−0·5‡	2·2
Control group	7·0	3·2	10·0*	6·5	3·0	4·0
Total cholesterol (mg/dl)‖						
Nutrition programme group	191·1	33·2	201·1	33·6	10·0	18·1
Control group	183·6	41·0	196·8*	48·1	13·2	15·9
HDL-cholesterol (mg/dl)‖						
Nutrition programme group	62·0	18·0	70·0*	21·0	8·0	10·0
Control group	55·0	19·0	58·0	21·0	3·0	5·0
Total cholesterol:HDL-cholesterol ratio						
Nutrition programme group	3·5	1·6	3·2*	1·5	−0·3‡	0·3
Control group	3·5	0·9	3·5	0·8	0·02	0·4
LDL-cholesterol (mg/dl)‖						
Nutrition programme group	111·4	37·3	116·6	44·3	5·2	18·3
Control group	111·7	29·9	121·1*	35·7	9·3	14·1
TAG (mg/dl)‖						
Nutrition programme group	91·9	46·6	70·5*	34·8	−21·4	28·5
Control group	85·1	40·7	87·1	40·3	2·0	29·8
HOMA2						
Nutrition programme group	0·91	0·45	0·73	0·49	−0·18§	0·27
Control group	0·93	0·43	1·33†	0·86	0·40	0·53

ALT, alanine aminotransferase; AST, aspartate aminotransferase; HOMA2, updated homoeostasis model assessment.

* Mean value was significantly different from that at baseline (*P* < 0·05; within group; paired Student's *t* test).

† Mean value was significantly different from that at baseline (*P* < 0·05; within group; Wilcoxon matched-pairs test).

‡ Mean value was significantly different from that of the control group (*P* < 0·05; between groups; unpaired Student's *t* test)

§ Mean value was significantly different from that of the control group (*P* < 0·05; between groups; Mann–Whitney *U* test).

‖ To convert insulin in *μ*IU/ml to pmol/l, multiply by 6·945. To convert cholesterol in mg/dl to mmol/l, multiply by 0·0259. To convert TAG in mg/dl to mmol/l, multiply by 0·0113.

### Adverse events

Although a total of sixty-nine adverse events were reported by all participants in the nutrition programme group, only ten (14·5 %) were possibly related to the dietary intervention. Those ten events, intermittent and gastrointestinal in nature, and of mild or moderate severity, were reported by two (20 %) subjects on fasting days. One subject reported flatulence on eight separate occasions over a total of 18 d. The other subject reported two separate episodes of nausea, the first lasting 13 d and the second lasting 2 d. Those events did not require any treatment and were ultimately resolved. No severe adverse events were reported. On the basis of these results, the nutrition programme appears overall safe and well tolerated.

## Discussion

This pilot study showed that a modified 5:2 nutrient-supported intermittent energy restriction nutrition programme might be a promising strategy to manage and prevent weight gain and support metabolic health during the winter holiday season in healthy overweight individuals. The compliance rate was very high, and no severe adverse events were reported, suggesting that this dietary intervention appears to be safe and well tolerated.

Subjects who followed the nutrition programme for 52 d during the winter holiday season not only did not gain weight, but also lost a modest yet significant 1·3 kg compared with their baseline pre-holiday weight. The results are in line with studies reporting the beneficial effects of various intermittent energy restriction interventions on body weight^(^^21^^,^^53^^–^^63^^)^. However, no between-group difference in weight loss after 52 d was observed, indicating that the study was underpowered. In contrast to prior studies conducted in the USA^(^^1^^–^^5^^)^, subjects in the control group who consumed their habitual holiday diet did not gain any weight. Although surprising, this could in part be explained by subjects being inclined to slightly change their food choices, and therefore their energy intake, knowing they were participating in a weight management study. Similar results were reported by others in adults^(^^64^^)^ and college students^(^^65^^)^ between Thanksgiving and New Year. Results also indicated the beneficial effects of 2-d energy restriction on key metabolic markers in subjects enrolled in the nutrition programme. In participants in the control group who consumed their holiday diet between Thanksgiving and New Year, fasting insulin and total and LDL-cholesterol levels significantly increased compared with normal range values at baseline. HOMA2 also increased, indicative of greater insulin resistance. Overindulgence of festive foods, often rich in carbohydrates and fat, may have negatively impacted metabolic health, probably increasing insulin requirements and lipid levels^(^^6^^)^. Those effects are detrimental, as insulin resistance is associated with a cluster of metabolic disorders, including dyslipidaemia, and is a risk factor for CVD^(^^66^^)^. In contrast, while still consuming their holiday festive food *ad libitum* 5 d a week, subjects who followed the nutrition programme experienced a significant reduction in fasting TAG and an increase in HDL-cholesterol levels compared with the pre-holiday baseline. Worthy of attention is the fact that although overweight, these subjects had a lipid profile within normal range at the beginning of the study and therefore were unlikely to benefit from substantial improvements in serum metabolic parameters. However, these predictable acute changes are potentially due to the 2-d energy restriction rather than the nutrition programme itself^(^^22^^,^^67^^)^, and might normalise within a few days of resuming habitual energy intake. Although likely to be transient, these positive changes from fasting remain clinically meaningful, as maintaining an optimal lipid profile is known to be critical to promote cardiovascular health^(^^68^^)^. Long-term consequences of these changes are yet to be investigated, but, if repeated, these effects might help maintain healthy TAG and HDL-cholesterol levels already within normal range in healthy individuals. We did not observe any reduction in fasting insulin level resulting from 2-d intermittent energy restriction in subjects in the nutrition programme group^(^^22^^)^. However, we observed a significant between-group difference in changes from baseline in fasting insulin levels, although it was mainly driven by the aforementioned increase in insulin levels in the control group. Between-group differences were also significant for total cholesterol:HDL-cholesterol ratio and HOMA2, suggesting beneficial effects of the intermittent energy restriction intervention on known risk factors for CVD.

To our knowledge, there is little research on 5:2 dietary interventions, and none conducted during the winter holiday period. In a 6-month randomised study, Harvie *et al*.^(^^22^^)^ described the benefits of a 2-d/week energy restriction intervention (about 650 kcal/d; 2720 kJ/d) on body weight, total cholesterol, LDL-cholesterol, TAG, fasting insulin and insulin sensitivity in young overweight or obese women. In another study, obese male adults who consumed a 5:2 diet with 2 d of 600 kcal/d (2510 kJ/d) and 5 d of habitual eating per week for 6 months experienced significant weight loss^(^^24^^)^. And, in a third study, Sundfør *et al*.^(^^29^^)^ reported improvements in TAG and HDL-cholesterol and weight loss in obese adults engaged in intermittent energy restriction with 400–600 kcal/d (1670–2510 kJ/d) on 2 d per week for 6 months. Although limited, these data highlight the significance of 5:2 dietary strategies to support weight loss and metabolic health.

### Strengths and limitations of the study

One strength of the present study is that this is the first randomised clinical trial to investigate the efficacy of a modified 5:2 nutrition plan combined with dietary supplements for weight management during the winter holiday season. Another strength is the very high compliance rate. Adherence to conventional weight-loss programmes such as energy restriction interventions in human subjects is notoriously low^(^^69^^)^, especially during the holiday season. The very high compliance rate reported in this study indicates that participants did not have any difficulty adhering to this intermittent energy restriction nutrition programme in a real-life situation during the holiday season. One reason might be that this nutrition programme has the advantage over other dietary interventions for weight management of being achievable, simple and easy to follow, with no ‘calorie counting’ or food restrictions 5 d per week. In addition, participants consumed a balanced shake combined with dietary supplements to support weight management efforts, which were likely to help with compliance. This plan was tested among healthy overweight individuals who were most likely to benefit from a real-life dietary intervention and motivated to lose weight.

This pilot study had several limitations. First, this single-centre study used a small convenience sample, mainly from Life Extension employees, and not a population-based sample. Therefore, subjects who participated in this trial might have been more health conscious and more engaged in weight-loss efforts knowing they were part of a weight management study. For this reason, the results may lack external validity and may not be applicable to the general population. Future studies on a larger cohort of participants recruited from the whole population are needed to confirm the study findings. The second limitation was an imbalance in the number of participants between both groups, which may be due to the block size. Third, serum metabolic markers were measured in the morning after the two fasting days in the nutrition programme group. In future studies, 12-h fasting blood samples should be collected after 5 d of *ad libitum* energy intake to ascertain effects of the nutrition programme on serum metabolic markers. Fourth, the absence of a between-group difference in body weight changes after 52 d indicated that the nutrition programme did not have an overall effect compared with the control group, and that the study was underpowered. After conducting a *post hoc* power analysis, it was determined that enrolment of twenty subjects per group would provide 80 % power to detect a between-group difference of 1·36 kg at a two-sided 0·05 level of significance with an estimated standard deviation of 1·5 kg. Fifth, participants were not required to self-report their food intake describing their dietary pattern on fed days during the holiday season. They did not have to log their physical activity either. Self-reported data can be inherently biased and imprecise and needs to be interpreted with caution^(^^70^^)^. However, validated self-reporting tools can provide a valuable piece of information about energy intake and expenditure during non-fasting days. Sixth, the study did not include a survey to determine how participants felt about the nutrition programme, if they would recommend it to others, and if they would be likely to use it again. Finally, the study lacked validated questionnaires to evaluate the impact of the nutrition programme on various aspects of participants’ lives, including sleep, mood and quality of life.

Taken together, results from this pilot study suggest this modified 5:2 intermittent energy restriction nutrition programme is a promising dietary strategy to support weight management in healthy overweight adults during the winter holiday season. Additional research in a larger sample is warranted to assess long-term adherence and effectiveness of this nutrition plan and confirm these encouraging findings.

## References

[1] Yanovski JA, Yanovski SZ, Sovik KN, (2000) A prospective study of holiday weight gain. N Engl J Med 342, 861–867. 10727591 10.1056/NEJM200003233421206 PMC4336296

[2] Stevenson JL, Krishnan S, Stoner MA, (2013) Effects of exercise during the holiday season on changes in body weight, body composition and blood pressure. Eur J Clin Nutr 67, 944–949. 23695203 10.1038/ejcn.2013.98

[3] Andersson I & Rossner S (1992) The Christmas factor in obesity therapy. Int J Obes Relat Metab Disord 16, 1013–1015. 1335971

[4] Hull HR, Radley D, Dinger MK, (2006) The effect of the Thanksgiving holiday on weight gain. Nutr J 5, 29. 17118202 10.1186/1475-2891-5-29 PMC1660573

[5] Helander EE, Wansink B & Chieh A (2016) Weight gain over the holidays in three countries. N Engl J Med 375, 1200–1202. 10.1056/NEJMc1602012 27653588

[6] Rees SG, Holman RR & Turner RC (1985) The Christmas feast. Br Med J (Clin Res Ed) 291, 1764–1765. 10.1136/bmj.291.6511.1764 PMC1419184 3936575

[7] Reid R & Hackett AF (1999) Changes in nutritional status in adults over Christmas 1998. J Hum Nutr Diet 12, 513–516.

[8] Leung MY, Carlsson NP, Colditz GA, (2017) The burden of obesity on diabetes in the United States: Medical Expenditure Panel Survey, 2008 to 2012. Value Health 20, 77–84. 28212973 10.1016/j.jval.2016.08.735 PMC5319814

[9] Kachur S, Lavie CJ, de Schutter A, (2017) Obesity and cardiovascular diseases. Minerva Med 108, 212–228. 28150485 10.23736/S0026-4806.17.05022-4

[10] Himbert C, Delphan M, Scherer D, (2017) Signals from the adipose microenvironment and the obesity-cancer link – a systematic review. Cancer Prev Res *(*Phila*)* 10, 494–506. 28864539 10.1158/1940-6207.CAPR-16-0322 PMC5898450

[11] Kovesdy CP, Furth SL, Zoccali C, (2017) Obesity and kidney disease: hidden consequences of the epidemic. Can J Kidney Health Dis 4, 2054358117698669. 28540059 10.1177/2054358117698669 PMC5433675

[12] Abdelaal M, le Roux CW & Docherty NG (2017) Morbidity and mortality associated with obesity. Ann Transl Med 5, 161. 28480197 10.21037/atm.2017.03.107 PMC5401682

[13] Taylor VH, Forhan M, Vigod SN, (2013) The impact of obesity on quality of life. Best Pract Res Clin Endocrinol Metab 27, 139–146. 23731876 10.1016/j.beem.2013.04.004

[14] Wing RR, Lang W, Wadden TA, (2011) Benefits of modest weight loss in improving cardiovascular risk factors in overweight and obese individuals with type 2 diabetes. Diabetes Care 34, 1481–1486. 21593294 10.2337/dc10-2415 PMC3120182

[15] Magkos F, Fraterrigo G, Yoshino J, (2016) Effects of moderate and subsequent progressive weight loss on metabolic function and adipose tissue biology in humans with obesity. Cell Metab 23, 591–601. 26916363 10.1016/j.cmet.2016.02.005 PMC4833627

[16] Watras AC, Buchholz AC, Close RN, (2007) The role of conjugated linoleic acid in reducing body fat and preventing holiday weight gain. Int J Obes (Lond) 31, 481–487. 16924272 10.1038/sj.ijo.0803437

[17] Boutelle KN, Kirschenbaum DS, Baker RC, (1999) How can obese weight controllers minimize weight gain during the high risk holiday season? By self-monitoring very consistently. Health Psychol 18, 364–368. 10431937 10.1037//0278-6133.18.4.364

[18] Golbidi S, Daiber A, Korac B, (2017) Health benefits of fasting and caloric restriction. Curr Diab Rep 17, 123. 29063418 10.1007/s11892-017-0951-7

[19] Fontana L (2008) Calorie restriction and cardiometabolic health. Eur J Cardiovasc Prev Rehabil 15, 3–9. 18277179 10.1097/HJR.0b013e3282f17bd4

[20] Bales CW & Kraus WE (2013) Caloric restriction: implications for human cardiometabolic health. J Cardiopulm Rehabil Prev 33, 201–208. 23748374 10.1097/HCR.0b013e318295019e PMC3696577

[21] Varady KA, Bhutani S, Klempel MC, (2013) Alternate day fasting for weight loss in normal weight and overweight subjects: a randomized controlled trial. Nutr J 12, 146. 24215592 10.1186/1475-2891-12-146 PMC3833266

[22] Harvie MN, Pegington M, Mattson MP, (2011) The effects of intermittent or continuous energy restriction on weight loss and metabolic disease risk markers: a randomized trial in young overweight women. Int J Obesity (Lond) 35, 714–727. 10.1038/ijo.2010.171 PMC3017674 20921964

[23] Kim KH, Kim YH, Son JE, (2017) Intermittent fasting promotes adipose thermogenesis and metabolic homeostasis via VEGF-mediated alternative activation of macrophage. Cell Res 27, 1309–1326. 29039412 10.1038/cr.2017.126 PMC5674160

[24] Conley M, Le Fevre L, Haywood C, (2018) Is two days of intermittent energy restriction per week a feasible weight loss approach in obese males? A randomised pilot study. Nutr Diet 75, 65–72. 28791787 10.1111/1747-0080.12372

[25] Sutton EF, Beyl R, Early KS, (2018) Early time-restricted feeding improves insulin sensitivity, blood pressure, and oxidative stress even without weight loss in men with prediabetes. Cell Metab 27, 1212–1221.e1213. 29754952 10.1016/j.cmet.2018.04.010 PMC5990470

[26] Witte AV, Fobker M, Gellner R, (2009) Caloric restriction improves memory in elderly humans. Proc Natl Acad Sci U S A 106, 1255–1260. 19171901 10.1073/pnas.0808587106 PMC2633586

[27] Cignarella F, Cantoni C, Ghezzi L, (2018) Intermittent fasting confers protection in CNS autoimmunity by altering the gut microbiota. Cell Metab 27, 1222–1235.e1226. 29874567 10.1016/j.cmet.2018.05.006 PMC6460288

[28] Wei M, Brandhorst S, Shelehchi M, (2017) Fasting-mimicking diet and markers/risk factors for aging, diabetes, cancer, and cardiovascular disease. Sci Transl Med 9, eaai8700. 10.1126/scitranslmed.aai8700 PMC6816332 28202779

[29] Sundfør TM, Svendsen M & Tonstad S (2018) Effect of intermittent versus continuous energy restriction on weight loss, maintenance and cardiometabolic risk: a randomized 1-year trial. Nutr Metab Cardiovasc Dis 28, 698–706. 29778565 10.1016/j.numecd.2018.03.009

[30] Howe P & Buckley J (2014) Metabolic health benefits of long-chain omega-3 polyunsaturated fatty acids. Mil Med 179, 11 Suppl., 138–143. 25373098 10.7205/MILMED-D-14-00154

[31] Filipovic MG, Aeschbacher S, Reiner MF, (2018) Whole blood omega-3 fatty acid concentrations are inversely associated with blood pressure in young, healthy adults. J Hypertens 36, 1548–1554. 29570511 10.1097/HJH.0000000000001728 PMC6085127

[32] Kamal-Eldin A, Moazzami A & Washi S (2011) Sesame seed lignans: potent physiological modulators and possible ingredients in functional foods & nutraceuticals. Recent Pat Food Nutr Agric 3, 17–29. 21114470 10.2174/2212798411103010017

[33] Poudyal H, Campbell F & Brown L (2010) Olive leaf extract attenuates cardiac, hepatic, and metabolic changes in high carbohydrate-, high fat-fed rats. J Nutr 140, 946–953. 20335636 10.3945/jn.109.117812

[34] Rosato V, Temple NJ, La Vecchia C, (2017) Mediterranean diet and cardiovascular disease: a systematic review and meta-analysis of observational studies. Eur J Nutr (epublication ahead of print version 25 November 2017). 10.1007/s00394-017-1582-0 29177567

[35] Lyon MR & Kacinik V (2012) Is there a place for dietary fiber supplements in weight management? Curr Obes Rep 1, 59–67. 22611521 10.1007/s13679-012-0016-9 PMC3342503

[36] Celleno L, Tolaini MV, D'Amore A, (2007) A dietary supplement containing standardized *Phaseolus vulgaris* extract influences body composition of overweight men and women. Int J Med Sci 4, 45–52. 17299581 10.7150/ijms.4.45 PMC1796956

[37] Wu X, Xu X, Shen J, (2010) Enhanced weight loss from a dietary supplement containing standardized *Phaseolus vulgaris* extract in overweight men and women. J Appl Res 10, 73–79.

[38] Hausenblas HA, Heekin K, Mutchie HL, (2015) A systematic review of randomized controlled trials examining the effectiveness of saffron (*Crocus sativus* L.) on psychological and behavioral outcomes. J Integr Med 13, 231–240. 26165367 10.1016/S2095-4964(15)60176-5 PMC5747362

[39] Hidalgo J, Flores C, Hidalgo MA, (2014) Delphinol^®^ standardized maqui berry extract reduces postprandial blood glucose increase in individuals with impaired glucose regulation by novel mechanism of sodium glucose cotransporter inhibition. Panminerva Med 56, 2 Suppl. 3, 1–7. 24861886

[40] Rojo LE, Ribnicky D, Logendra S, (2012) *In vitro* and *in vivo* anti-diabetic effects of anthocyanins from maqui berry (*Aristotelia chilensis*). Food Chem 131, 387–396. 26279603 10.1016/j.foodchem.2011.08.066 PMC4535716

[41] Alvarado JL, Leschot A, Olivera-Nappa A, (2016) Delphinidin-rich maqui berry extract (Delphinol^®^) lowers fasting and postprandial glycemia and insulinemia in prediabetic individuals during oral glucose tolerance tests. BioMed Res Int 2016, 9070537. 28025651 10.1155/2016/9070537 PMC5153493

[42] Kato M, Tani T, Terahara N, (2015) The anthocyanin delphinidin 3-rutinoside stimulates glucagon-like peptide-1 secretion in murine GLUTag cell line via the Ca^2+^/calmodulin-dependent kinase II pathway. PLOS ONE 10, e0126157. 25962102 10.1371/journal.pone.0126157 PMC4427495

[43] Marin YE, Wall BA, Wang S, (2007) Curcumin downregulates the constitutive activity of NF-κB and induces apoptosis in novel mouse melanoma cells. Melanoma Res 17, 274–283. 17885582 10.1097/CMR.0b013e3282ed3d0e

[44] Park C, Moon DO, Choi IW, (2007) Curcumin induces apoptosis and inhibits prostaglandin E_2_ production in synovial fibroblasts of patients with rheumatoid arthritis. Int J Mol Med 20, 365–372. 17671742

[45] Noorafshan A & Ashkani-Esfahani S (2013) A review of therapeutic effects of curcumin. Curr Pharm Des 19, 2032–2046. 23116311

[46] Bhattacharyya S, Pal D, Banerjee D, (2009) Shilajit dibenzo-*α*-pyrones: mitochondria targeted antioxidants. Pharmacologyonline 2, 690–698.

[47] Mishra RN & Joshi D (2011) Jiao Gu Lan (*Gynostemma pentaphyllum*): the Chinese Rasayan – current research scenario. Int J Res Pharm Biomed Sci 2, 1483–1502.

[48] Park SH, Huh TL, Kim SY, (2014) Antiobesity effect of *Gynostemma pentaphyllum* extract (actiponin): a randomized, double-blind, placebo-controlled trial. Obesity *(*Silver Spring*)* 22, 63–71. 23804546 10.1002/oby.20539

[49] Rizza S, Muniyappa R, Iantorno M, (2011) Citrus polyphenol hesperidin stimulates production of nitric oxide in endothelial cells while improving endothelial function and reducing inflammatory markers in patients with metabolic syndrome. J Clin Endocrinol Metab 96, E782–E792. 21346065 10.1210/jc.2010-2879 PMC3085197

[50] Levy JC, Matthews DR & Hermans MP (1998) Correct homeostasis model assessment (HOMA) evaluation uses the computer program. Diabetes Care 21, 2191–2192. 9839117 10.2337/diacare.21.12.2191

[51] Hill NR, Levy JC & Matthews DR (2013) Expansion of the homeostasis model assessment of β-cell function and insulin resistance to enable clinical trial outcome modeling through the interactive adjustment of physiology and treatment effects: iHOMA2. Diabetes Care 36, 2324–2330. 23564921 10.2337/dc12-0607 PMC3714535

[52] Song YS, Hwang YC, Ahn HY, (2016) Comparison of the usefulness of the updated homeostasis model assessment (HOMA2) with the original HOMA1 in the prediction of type 2 diabetes mellitus in Koreans. Diabetes Metab J 40, 318–325. 27273908 10.4093/dmj.2016.40.4.318 PMC4995187

[53] Bhutani S, Klempel MC, Kroeger CM, (2013) Alternate day fasting and endurance exercise combine to reduce body weight and favorably alter plasma lipids in obese humans. Obesity *(*Silver Spring*)* 21, 1370–1379. 23408502 10.1002/oby.20353

[54] Eshghinia S & Mohammadzadeh F (2013) The effects of modified alternate-day fasting diet on weight loss and CAD risk factors in overweight and obese women. J Diabetes Metab Disord 12, 4. 23497604 10.1186/2251-6581-12-4 PMC3598220

[55] Hoddy KK, Gibbons C, Kroeger CM, (2016) Changes in hunger and fullness in relation to gut peptides before and after 8 weeks of alternate day fasting. Clin Nutr 35, 1380–1385. 27062219 10.1016/j.clnu.2016.03.011

[56] Alhamdan BA, Garcia-Alvarez A, Alzahrnai AH, (2016) Alternate-day versus daily energy restriction diets: which is more effective for weight loss? A systematic review and meta-analysis. Obes Sci Pract 2, 293–302. 27708846 10.1002/osp4.52 PMC5043510

[57] Harris L, Hamilton S, Azevedo LB, (2018) Intermittent fasting interventions for treatment of overweight and obesity in adults: a systematic review and meta-analysis. JBI Database System Rev Implement Rep 16, 507–547. 10.11124/JBISRIR-2016-003248 29419624

[58] Klempel MC, Kroeger CM, Bhutani S, (2012) Intermittent fasting combined with calorie restriction is effective for weight loss and cardio-protection in obese women. Nutr J 11, 98. 23171320 10.1186/1475-2891-11-98 PMC3511220

[59] Gabel K, Hoddy KK, Haggerty N, (2018) Effects of 8-hour time restricted feeding on body weight and metabolic disease risk factors in obese adults: a pilot study. Nutr Healthy Aging 4, 345–353. 29951594 10.3233/NHA-170036 PMC6004924

[60] Byrne NM, Sainsbury A, King NA, (2018) Intermittent energy restriction improves weight loss efficiency in obese men: the MATADOR study. Int J Obes (Lond) 42, 129–138. 28925405 10.1038/ijo.2017.206 PMC5803575

[61] Antoni R, Johnston KL, Collins AL, (2018) Intermittent *v.* continuous energy restriction: differential effects on postprandial glucose and lipid metabolism following matched weight loss in overweight/obese participants. Br J Nutr 119, 507–516. 29508693 10.1017/S0007114517003890

[62] Ash S, Reeves MM, Yeo S, (2003) Effect of intensive dietetic interventions on weight and glycaemic control in overweight men with type II diabetes: a randomised trial. Int J Obes Relat Metab Disord 27, 797–802. 12821964 10.1038/sj.ijo.0802295

[63] Williams KV, Mullen ML, Kelley DE, (1998) The effect of short periods of caloric restriction on weight loss and glycemic control in type 2 diabetes. Diabetes Care 21, 2–8. 9538962 10.2337/diacare.21.1.2

[64] Wagner DR, Larson JN & Wengreen H (2012) Weight and body composition change over a six-week holiday period. Eat Weight Disord 17, e54–e56. 22751272 10.1007/BF03325328

[65] Hull HR, Hester CN & Fields DA (2006) The effect of the holiday season on body weight and composition in college students. Nutr Metab 3, 44. 10.1186/1743-7075-3-44 PMC1766354 17192197

[66] Ormazabal V, Nair S, Elfeky O, (2018) Association between insulin resistance and the development of cardiovascular disease. Cardiovasc Diabetol 17, 122. 30170598 10.1186/s12933-018-0762-4 PMC6119242

[67] Horne BD, Muhlestein JB, Lappe DL, (2013) Randomized cross-over trial of short-term water-only fasting: metabolic and cardiovascular consequences. Nutr Metab Cardiovasc Dis 23, 1050–1057. 23220077 10.1016/j.numecd.2012.09.007

[68] Ference BA, Graham I, Tokgozoglu L, (2018) Impact of lipids on cardiovascular health: *JACC* Health Promotion Series. J Am Coll Cardiol 72, 1141–1156. 30165986 10.1016/j.jacc.2018.06.046

[69] Dansinger ML, Gleason JA, Griffith JL, (2005) Comparison of the Atkins, Ornish, Weight Watchers, and Zone diets for weight loss and heart disease risk reduction: a randomized trial. JAMA 293, 43–53. 15632335 10.1001/jama.293.1.43

[70] Subar AF, Freedman LS, Tooze JA, (2015) Addressing current criticism regarding the value of self-report dietary data. J Nutr 145, 2639–2645. 26468491 10.3945/jn.115.219634 PMC4656907

